# Clinical Trial Designs for Rare Disorders: A Scoping Review of the Effectiveness of Pharmacologic Interventions in Fragile X Syndrome

**DOI:** 10.1212/NXG.0000000000200373

**Published:** 2026-03-24

**Authors:** Adam Van Steenbergen, Manpreet Kaur, Keneizha Rubanarayana, Francois Bolduc

**Affiliations:** From the Department of Pediatrics, University of Alberta, Edmonton, Canada.

## Abstract

**Purpose of Review:**

Rare disorders (RDs) collectively affect a substantial proportion of the population, yet most lack clinically approved, mechanistically targeted therapies. Fragile X syndrome (FXS), the most common inherited cause of intellectual disability and a major genetic contributor to autism spectrum disorder, exemplifies this gap. Despite more than 3 decades of preclinical research and numerous pharmacologic clinical trials informed by disease mechanisms, no targeted therapy for FXS has received regulatory approval. This scoping review aims to evaluate the design characteristics, outcome measures, and reported effect sizes of pharmacologic clinical trials in FXS to highlight methodological limitations that may have hindered the accurate assessment of therapeutic efficacy in FXS and to inform future trials in RDs.

**Recent Findings:**

We conducted a scoping review of published interventional studies involving individuals with FXS, identified through searches of PubMed, Excerpta Medica Database (EMBASE), PsycINFO, and CENTRAL. Eligible studies were pharmacologic trials that reported sufficient statistical information to calculate effect sizes for recurrent behavioral outcome measures. 23 trials met inclusion criteria, comprising 14 placebo-controlled randomized controlled trials (RCTs) and 9 open-label studies, with a total of 1,469 participants. Recurrent outcome measures included the Aberrant Behavior Checklist (the FXS-specific ABC-Cfx), the Clinical Global Impressions–Improvement scale, the Vineland Adaptive Behavior Scales–Second Edition (VABS-II), and the Visual Analog Scale (VAS) for Anxiety (VAS Anxiety). The median absolute effect sizes were small among RCTs (|d| = 0.22) and moderate among open-label studies (|d| = 0.70). Across studies, substantial heterogeneity was observed in trial design, sample size, participant characteristics, and outcome measures, complicating cross-trial comparisons and interpretation of efficacy signals.

**Summary:**

Collectively, existing pharmacologic trials in FXS have demonstrated limited and inconsistent treatment effects, with interpretation constrained by small sample sizes, heterogeneity in designs and outcome measures, and differences in participant characteristics. Greater standardization of trial design and outcome assessment, improved alignment between outcome measures and treatment mechanisms, and careful consideration of participant stratification are critical to improving signal detection in future trials. These insights have broader relevance for RD research, where methodological rigor is essential to translating promising mechanisms into effective therapies.

## Introduction

Rare disorders (RDs) are diverse conditions with a global prevalence of 5–80 per 100,000 people.^1^ RDs significantly affect individuals and society. Their low prevalence and complexity make clinical testing difficult, even with strong preclinical evidence.

Many RDs are genetic, present in childhood, and accompanied by neurodevelopmental disabilities (NDDs) such as autism spectrum disorder (ASD) and intellectual disability (ID). Fragile X syndrome (FXS), the leading inherited cause of both ASD and ID, results from a Cytosine-Guanine-Guanine (CGG) trinucleotide expansion in the fragile X mental retardation^1^ (*FMR1*) gene, which silences production of fragile X mental retardation protein (FMRP).^2,3^ Despite decades of research and multiple clinical trials, no approved mechanistic treatment exists, and heterogeneity in symptom expression likely contributes to variable treatment response.^4,5^

Therefore, we aimed to compare pharmacologic trials in individuals with FXS by their key design characteristics (study design, outcome measures, sample size) and the reported efficacy of the investigational product. To enable these comparisons, we calculated effect sizes for outcome measures that overlapped across trials.

This scoping review aimed to compile the pharmacologic interventions tested in human FXS trials, summarize the overlapping outcome measures, and evaluate the magnitude of treatment effects. A further goal was to contrast placebo-controlled and open-label studies to assess the effect of study design on effect size. Finally, we aimed to identify areas for improvement in trial design. Our research questions were as follows:

1. What pharmacologic agents have been tested in human FXS trials?
2. Which outcome measures recur across trials, and how consistently are they reported?
3. What is the magnitude and direction of treatment effects on these outcome measures?
4. How do effect sizes differ between randomized, placebo-controlled trials and open-label studies?
5. Where are the most critical design weaknesses?

Across FXS clinical trials, a small set of behavioral outcomes function as de facto “field standards” for detecting change. Caregiver-reported and clinician-reported scales, most commonly the Aberrant Behavior Checklist-Community (ABC-C) and its FXS-specific version (ABC-Cfx), the Clinical Global Impressions–Improvement (CGI-I), the Vineland Adaptive Behavior Scales (VABS; versions II/III), and Visual Analog Scale (VAS) for Anxiety (VAS Anxiety), are the most frequently deployed and, therefore, the most comparable across studies. These measures are widely used because they are feasible across age and ability levels and are familiar to clinicians and families.^6,7^

In this review, we concentrated on these overlapping caregiver-reported and clinician-reported outcomes, which are typically specified as secondary end points and can be vulnerable to expectancy, rater context effects, and limited sensitivity to cognitive or neurobiological targets.^6,7^ This approach maximizes comparability but also means the resulting effect sizes reflect changes in these reported domains rather than overall investigational product efficacy.

Important classes of outcomes were not the focus of this review because they were insufficiently recurrent across trials to support pooled effect size estimation. These include (1) performance-based cognitive batteries (e.g., NIH Toolbox Cognition Battery, KiTAP), (2) objective/physiologic or neurobiological markers (e.g., EEG/ Event-Related Potential (ERP) features, eye-tracking, autonomic indices), and (3) assay-based or biomarker end points (e.g., pharmacodynamic readouts). These domains can offer improved construct validity and mechanistic specificity, but their current use in FXS trials is heterogeneous (different tasks, scoring metrics, and age bands), limiting cross-study synthesis. By contrast, caregiver-reported scales are prominent in both interventional and natural history/registry efforts and provide continuity for longitudinal comparison, albeit with the subjectivity costs noted above.^7^

## Methods

This scoping review was conducted per PRISMA-ScR (Preferred Reporting Items for Systematic Reviews and Meta-Analyses extension for Scoping Reviews) guidelines. A retrospective protocol for this scoping review was registered on the Open Science Framework to enhance transparency.^8^

We conducted a PubMed search using the search string “Fragile X syndrome” OR “FXS” to identify peer-reviewed clinical trials. We applied the “Clinical Trial” filter on PubMed and downloaded the list of publication IDs. PubMed's application programming interface (API) was then used to collect the abstracts' content into a spreadsheet for ease of data organization. In addition, we searched Excerpta Medica Database (Embase), PsycINFO, and Central using the “Fragile X syndrome” OR “FXS” search query and then applied a filter to find clinical trials. The search was last conducted in April 2025 with no date restrictions. In total, 289 studies were identified.

Studies were eligible if they were randomized placebo-controlled trials or open-label clinical trials involving human participants with FXS, were published in English, and reported overlapping outcome measures suitable for comparison. To enable effect size calculation, publications had to report, or provide sufficient data to derive, the mean change from baseline, corresponding SDs, and sample sizes for each outcome measure in treatment and placebo groups. When data were unavailable, authors were contacted.

After identifying and removing duplicate studies, 2 reviewers independently screened the titles and abstracts, with a third reviewer resolving any conflicts.

Data were extracted using a standardized spreadsheet form capturing study titles, presence of placebo, outcome measures, and statistical data required for effect size calculation. The form was piloted for clarity and applied by 1 reviewer, with independent verification by a second. Discrepancies were resolved by discussion and consensus.

Effect sizes (Cohen's d) were calculated as the difference in mean change scores between treatment and placebo groups, standardized by the pooled within-group SD to account for baseline differences.^9^ Standard errors and 95% CIs were computed for each estimate. Pooled analyses were conducted in Review Manager using a random-effects model with inverse-variance weighting to account for within- and between-study variability.^10^ The pooled effect size represents a weighted average of individual study effects with corresponding 95% CIs.^11^ Conventional thresholds were applied to interpret magnitude (small = 0.2, medium = 0.5, large ≥0.8).^12^

When change score SDs were unreported, they were estimated using standard formulas (eMethods). In the absence of reported correlations between baseline and follow-up measures, we assumed r = 0.7, consistent with previous FXS and autism meta-analyses using similar outcome measures.^13,14^ Sensitivity analyses using r = 0.5 and r = 0.9 were conducted to assess robustness. Detailed formulas and exceptions are provided in eMethods.

Effect sizes for the CGI-I scale could not be calculated for open-label studies because CGI-I is inherently a postbaseline global measure of change that does not have a baseline value. Unlike the CGI-Severity, which can be measured at multiple time points to derive pre-post differences, the CGI-I provides a single rating of improvement relative to baseline (ranging from “very much improved” to “very much worse”). As a result, standardized mean differences or change-from-baseline statistics cannot be computed, since these require 2 time-specific means.

Our primary analysis was restricted to outcome measures that recurred across trials and permitted standardized effect size estimation (e.g., ABC-C/ABC-Cfx, CGI-I, VAS Anxiety, VABS). In most trials, these were specified as secondary end points. Primary end points (e.g., safety, specific cognitive batteries, biomarker assays) were catalogued but not pooled unless they overlapped across studies. Accordingly, the effect sizes reported here are targeted to recurrent reported outcomes and are not intended to represent overall efficacy of each investigational compound.

### Standard Protocol Approvals, Registrations, and Patient Consents

This study is a scoping review of published clinical trials and did not involve any direct interaction with human participants or animals. Accordingly, institutional ethics approval and patient consent were not required. All analyzed data were derived from previously published studies that had obtained ethics approval from their respective institutions.

### Data Availability

All data in this review were derived from publicly available clinical trial reports and peer-reviewed publications. No individual participant-level data were collected. The data set of trial characteristics and calculated effect sizes are available from the corresponding author upon reasonable request for academic, noncommercial purposes. These materials will be made available beginning at the time of publication, with no planned end date. Requests should be submitted by email to the corresponding author.

## Results

**Figure 1:**
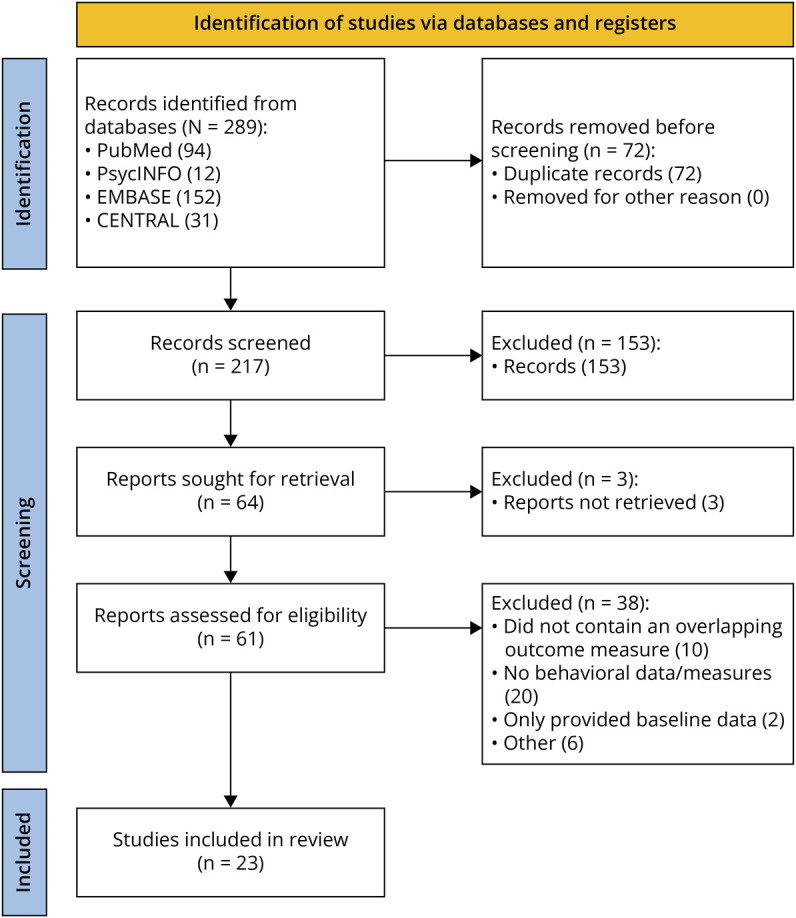
PRISMA 2020 Flow Diagram Outlining the Identification, Screening, Eligibility, and Inclusion of Studies in This Scoping review^50^ A total of 289 records were identified across 4 databases: PubMed (n = 94), PsycINFO (n = 12), Embase (n = 152), and Central (n = 31). After removing 72 duplicate records, 217 records were screened, with 64 full-text articles sought for retrieval. Reasons for exclusion included not containing a pharmacologic treatment (n = 61), not being about FXS (n = 75), not containing human participants (n = 3), and not being a clinical trial (n = 14). Ultimately, 23 studies met inclusion criteria. Reasons for full-text exclusion included: absence of overlapping outcome measures (n = 10), lack of behavioral outcome data (n = 20), provision of only baseline data (n = 2), full text not accessible (n = 3) or other reasons (n = 6).

The screening process is illustrated in the Preferred Reporting Items for Systematic Reviews and Meta-Analyses (PRISMA) flow diagram (Figure 1). Of the 217 studies screened at the title and abstract level, 153 were excluded for not meeting the inclusion criteria (e.g., not focused on FXS, lacking a treatment component). The remaining 64 studies underwent full-text screening. Of these, 41 were excluded due to lacking overlapping outcome measures, behavioral data, or only reporting baseline data.

This resulted in 23 studies eligible for inclusion in the scoping review. Fourteen of these included a placebo group and reported all necessary data and overlapping outcome measures for the primary analysis. Although the remaining 9 clinical trials did not contain placebo groups, they had effect sizes with overlapping outcome measures that may warrant further investigation. For these 9 studies, the effect size was calculated by comparing the means and SDs at baseline and follow-up.

**Figure 2:**
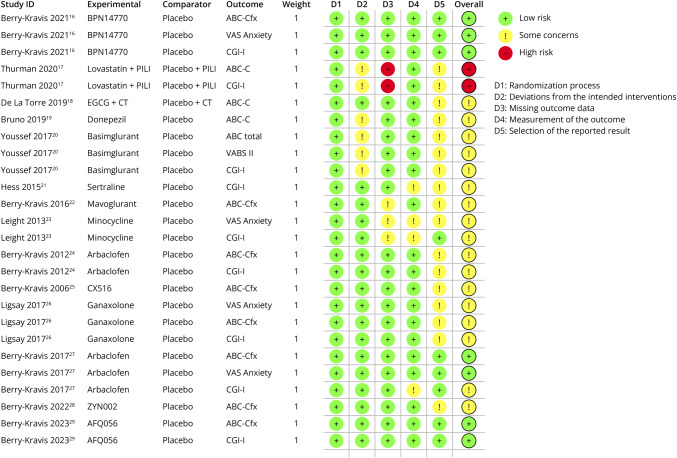
Risk of Bias Assessment Showing the Randomized Controlled Trials Included in This Scoping Review ABC-C = Aberrant Behavior Checklist–Community version; ABC-Cfx = Aberrant Behavior Checklist–Community, Fragile X version; CGI-I = Clinical Global Impressions–Improvement scale; D1 = Randomization process; D2 = Deviation from the intended interventions; D3 = Missing outcome data; D4 = Measurement of the outcome; D5 = Selection of the reported result; EGCG + CT = epigallocatechin-3-gallate and cognitive training; PILI = Parent-implemented language intervention; VABS-II = Vineland Adaptive Behavior Scales, Second Edition; VAS = Visual Analog Scale.

The risk of bias for the 14 randomized controlled trials (RCTs) was assessed using the Cochrane Risk of Bias 2 tool to contextualize the calculated effect sizes and improve the interpretability of outcomes across placebo-controlled trials (Figure 2).^15^ Open-label studies were not assessed due to the absence of a validated risk-of-bias tool for uncontrolled pharmacologic trials. Common concerns identified in domain 5 included a lack of a prespecified analysis plan, post hoc analyses, and limited transparency.

For each outcome measure used in more than 1 study, we generated forest plots to display the effect size of each individual trial. We also computed the overall effect size observed for all trials on that outcome measure to assess whether some outcome measures were showing more “movement.” Because the included trials evaluated different investigational products, these aggregated effect sizes reflect properties of the outcome measures themselves rather than drug-specific efficacy. This approach also enabled us to compare general trends between placebo-controlled and open-label trials.

The characteristics and outcome measures of the included placebo-controlled trials are summarized in Table 1, with detailed study design information provided in (eTable 1). Reported effect sizes primarily reflect secondary caregiver-reported or clinician-reported outcomes.

**Table 1:** Summary of Randomized Controlled Trials in Fragile X Syndrome That Included a Placebo Group and Overlapping Behavioral Outcome Measures Used for Effect Size Calculations

Study	Drug/Treatment	Study length	Population	Participants	n	Outcome measures
Berry-Kravis 2021 (study 1)^16^	BPN14770	24 wk	FXS	Adults	30	VAS anxiety, ABC-Cfx, CGI-I
Thurman 2020 (study 4)^17^	Lovastatin	20 wk	FXS	Youth	30	ABC-C total, CGI-I
De La Torre 2020 (study 6)^18^	Epigallocatechin-3-gallate	6 mo	FXS	Adults	27	ABC-C subscales
Bruno 2019 (study 8)^19^	Donepezil	12 wk	FXS	Adolescents and adults	42	ABC-C total, ABC-Cfx subscales
Youssef 2017 (study 15)^20^	Basimglurant	12 wk	FXS	Adolescents and adults	183	CGI-I, ABC-C total, VABS-II
Greiss Hess 2016 (study 19)^21^	Sertraline	6 mo	FXS	Children	52	CGI-I
Berry-Kravis 2016 (study 22)^22^	Mavoglurant	12 wk	FXS	Adolescents and adults	314	ABC-Cfx subscales, CGI-I
Leigh 2013 (study 34)^23^	Minocycline	6 mo (3 mo then switched to other treatment)	FXS	Children and adolescents	55	VAS anxiety, CGI-I
Berry-Kravis 2012 (study 37)^24^	Arbaclofen	8 wk (4 wk then switched to other treatment)	FXS	Children and adults	63	Vineland II, CGI-I, ABC-Cfx subscales
Berry-Kravis 2006 (study 69)^25^	CX516	4 wk	FXS	Adults	49	ABC-C subscales
Ligsay 2017 (study 102)^26^	Ganaxolone	10–12 wk	FXS	Children and adolescents	59	VAS anxiety, CGI-I, ABC-Cfx subscales
Berry-Kravis 2017 (study 104)^27^	Arbaclofen	8 wk	FXS	Adolescents and adults	125	ABC-Cfx subscales, CGI-I, VAS anxiety
Berry-Kravis 2022 (study 215)^28^	Transdermal cannabidiol	12 wk	FXS	Adolescents and children	212	ABC-Cfx subscales
Berry-Kravis 2023 (study 216)^29^	Mavoglurant	6 mo	FXS	Children	110	ABC-Cfx subscales, CGI-I

Abbreviations: ABC-Cfx = Aberrant Behavior Checklist–Community, Fragile X version; ABC-C = Aberrant Behavior Checklist–Community version; CGI-I = Clinical Global Impressions–Improvement scale; FXS = Fragile X Syndrome; RCT = randomized controlled trials; VAS = visual analog scale; VABS-II = vineland adaptive behavior scales, Second Edition.

Each study is listed with the drug/treatment evaluated, population studied, participant demographics, sample size, and behavioral outcome measures reported. Overlapping outcomes include ABC-C, ABC-Cfx, CGI-I, VAS, and VABS-II.

The most frequently used outcome measure was the ABC-C and ABC-Cfx subscales, which appeared in 12 of the 14 placebo-controlled studies that also reported the data necessary for effect size calculations. Corresponding forest plots are shown (ABC-C: eFigure 1 and 2; ABC-Cfx: eFigure 3 and 4).

Among the ABC-Cfx subscales, statistically significant improvements were observed only with Arbaclofen, suggesting limited evidence for consistent treatment efficacy across these domains (eFigure 3). Arbaclofen yielded a medium effect size for Socially Unresponsive/Lethargic (Cohen *d* = −0.67, 95% CI −1.03 to −0.31) and a large effect size for Social Avoidance (*d* = −1.16, 95% CI −1.73 to −0.58).

BPN14770 produced small-to-medium effect sizes for Stereotypy (*d* = −0.55, 95% CI −1.28 to 0.17), hyperactivity (*d* = −0.44, 95% CI −1.16 to 0.29), and inappropriate speech (*d* = −0.61, 95% CI −1.34 to 0.12), warranting further investigation in larger, adequately powered trials. These results suggest potential benefits but remain inconclusive, as the corresponding 95% CIs all crossed zero, indicating statistical uncertainty.

**Figure 3:**
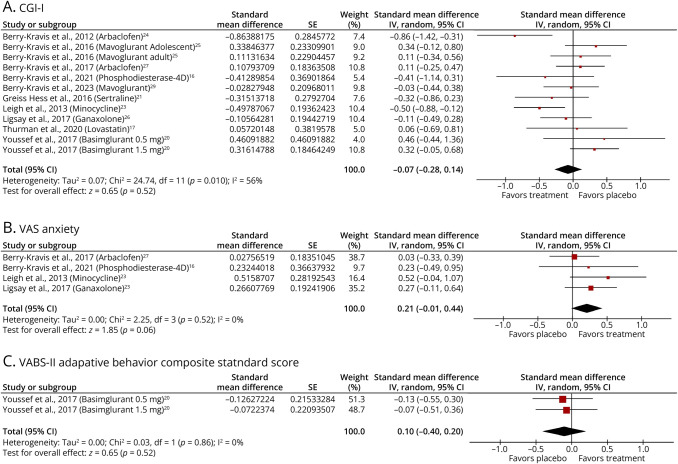
Forest Plots Showing the Effect Sizes and 95% CIs for the Effects of Pharmacologic Treatments Compared with Placebo on 3 Overlapping Outcome Measures in Individuals With FXS (A) Clinical Global Impressions–Improvement (CGI-I) scale. Negative effect size values indicate greater improvement in the treatment group. (B) Visual Analog Scale (VAS) for anxiety. Positive effect size values indicate a reduction in anxiety favoring treatment. (C) Vineland Adaptive Behavior Scales, Second Edition (VABS-II) Adaptive Behavior Composite scores. Negative effect size values indicate greater improvement in the placebo group. Only placebo-controlled trials with overlapping outcomes for each respective measure were included. FXS = Fragile X Syndrome.

For the CGI-I scale, Arbaclofen showed a large effect size (d = −0.86) in a 2012 phase 2 study, but this was not replicated in a 2017 phase 3 parallel-group trial (d = 0.11 in favour of placebo; Figure 3A).^24,27^ Differences in design, dosing flexibility, and participant characteristics, particularly younger, more impaired participants in phase 2, likely contributed to the divergent outcomes.

Minocycline also emerged as a promising treatment on the CGI-I, demonstrating a medium effect size (*d* = −0.50, 95% CI −0.88 to −0.12). Notably, it also reached statistical significance, as its 95% CI did not cross zero (−0.88 to −0.12). A phosphodiesterase-4D inhibitor also showed a negative effect size of −0.41, suggesting potential improvement. However, the wide CI (−1.14 to 0.31) renders the result inconclusive.

Among the 4 studies that assessed the VAS for Anxiety (VAS Anxiety), minocycline reported the largest effect size at 0.52 (Figure 3B). However, this result was not statistically significant, as the 95% CI includes zero (−0.04 to 1.07).

Both 0.5 mg and 1.5 mg of basimglurant were associated with small negative effect sizes for the Vineland Adaptive Behavior Scales–Second Edition (VABS-II) adaptive behavior composite standard score (−0.126 and −0.07, respectively), suggesting that the placebo group showed a greater improvement in symptoms compared with the treatment group (Figure 3C). However, these differences were not statistically significant.

Studies that did not include a placebo group but contained only a treatment group with overlapping outcome measures were analyzed and are presented in Table 2. In these open-label studies, a larger effect size was observed compared with placebo-controlled trials (median |*d*| = 0.70 for open-label studies vs 0.22 for placebo-controlled studies).

**Table 2:** Summary of Open-Label Clinical Trials in Fragile X Syndrome That Reported Overlapping Behavioral Outcome Measures but Did Not Include a Placebo Group

Study	Drug/treatment	Study design	Length of trial	Population	Participants	n	Outcome measures
Heussler 2019 (study 7)^30^	Transdermal cannabidiol	Phase 1/2, open-label, single-arm	12 wk	FXS	Children and adolescents	20	ABC-Cfx subscales and VAS anxiety
Caku 2014 (study 25)^31^	Lovastatin	Prospective, open-label, single-arm	12 wk	FXS	Children and adult males	15	ABC-Cfx subscales, VABS-II
Erickson 2013(study 31)^32^	Acamprosate	Prospective, open-label, single-arm	12 wk	FXS	Children and adolescents	12	ABC-Cfx subscales
Erickson 2011 (study 38)^33^	Aripiprazole	Prospective, open-label, single-arm	12 wk	FXS	Children, adolescents, and adults	12	ABC-C subscales
Erickson 2011 (study 40)^34^	Riluzole	Prospective, open-label, single-arm	6 wk	FXS	Adults	6	ABC-C subscales
Torrioli 2010 (study 41)^35^	Valproic acid	Open-label, single-arm, prospective	6 mo	FXS	Children and adolescent boys	10	VABS-II
Paribello 2010 (study 42)^36^	Minocycline	Prospective, open-label, add-on pilot trial	8 wk	FXS	Children, adolescents, and adults	20	ABC-C subscales
Kesler 2009 (study 45)^37^	Donepezil 5 mg and 10 mg	Open-label, single-arm pilot drug trial	6 wk	FXS	Adolescent and adult males	8	ABC-C subscales
Berry-Kravis 2008 (study 47)^38^	Lithium	Open-label, single-arm, prospective pilot clinical trial	2 mo	FXS	Children, adolescents, and adults	15	ABC-C subscales

Abbreviations: ABC-Cfx = Aberrant Behavior Checklist–Community, Fragile X version; ABC-C = Aberrant Behavior Checklist–Community version; CGI-I = Clinical Global Impressions–Improvement scale; FXS = Fragile X Syndrome; VAS = visual analog scale; VABS-II = vineland adaptive behavior scales, Second Edition.

Studies were included for effect size comparisons. Each study is listed with the drug or treatment tested, study design, participant demographics, sample size, and outcome measures reported. Overlapping outcomes include ABC-C, ABC-Cfx, VAS anxiety, and VABS-II.

**Figure 4:**
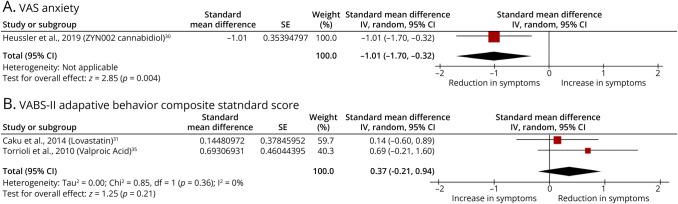
Forest Plots Showing the Effect Sizes and 95% CIs for the Effects of Pharmacologic Treatments on 2 Outcome Measures From Open-Label Studies Lacking a Placebo Group in Individuals With FXS Abbreviation: FXI = Fragile X syndrome. (A) Visual Analog Scale (VAS) for anxiety. Negative effect size values indicate a reduction in anxiety symptoms. (B) Vineland Adaptive Behavior Scales, Second Edition (VABS-II) Adaptive Behavior Composite scores. Positive effect size values indicate improvements in adaptive behavior. Only open-label trials reporting overlapping outcome measures for each respective scale were included.

For VAS Anxiety, transdermal cannabidiol (ZYN002) demonstrated a large, statistically significant effect size (*d* = −1.01, 95% CI −1.70 to −0.32), suggesting a reduction in anxiety among pediatric patients with FXS (Figure 4A). This promising finding highlights the need for further research into cannabidiol-based treatments for Fragile X–related anxiety, particularly through placebo-controlled trials.

A noteworthy finding was that valproic acid demonstrated a moderate to large effect size (*d* = 0.69, 95% CI −0.21 to 1.60) on the VABS-II Adaptive Behavior Composite Score, suggesting an improvement in adaptive functioning (communication, daily living skills, socialization, and motor skills) relative to baseline (Figure 4B). However, this result was not statistically significant, likely due to the small sample size (n = 10). Among the subdomains, the greatest improvement was observed in the Communication domain, which reported an effect size of 0.59. These preliminary findings warrant further investigation in larger, placebo-controlled studies to confirm their potential clinical relevance.

For the ABC-Cfx subscales, transdermal ZYN002 cannabidiol showed promising results (eFigure 4). Cannabidiol may alleviate various FXS symptoms by modulating the endocannabinoid system in the absence of FMRP.^28^ Medium-to-large effect sizes were observed across all subscales: −0.87 for Inappropriate Speech, −0.69 for Irritability, −0.76 for Socially Unresponsive/Lethargic, −0.59 for Hyperactivity, −1.00 for Social Avoidance, and −0.99 for Stereotypy. All effect sizes were statistically significant based on their 95% CIs, except for the Hyperactivity subscale.

Another notable finding was that lovastatin reported large effect sizes for Stereotypy (−1.04) and Social Avoidance (−0.96), while showing more modest effects for other subscales, such as Inappropriate Speech (−0.09). This suggests that lovastatin may be particularly effective in reducing symptoms related to Stereotypy and Social Avoidance in individuals with FXS. However, the effect size for lovastatin on the ABC-Cfx total score was 0, despite these large subscale-level effects. This discrepancy implies that improvements in specific domains may have been offset by unchanged or worsening behaviors in others. Although a *p* value of 0.05 was reported for the ABC-Cfx total score, indicating possible improvement, the median and interquartile range remained unchanged, limiting the interpretability of this result.

**Figure 5:**
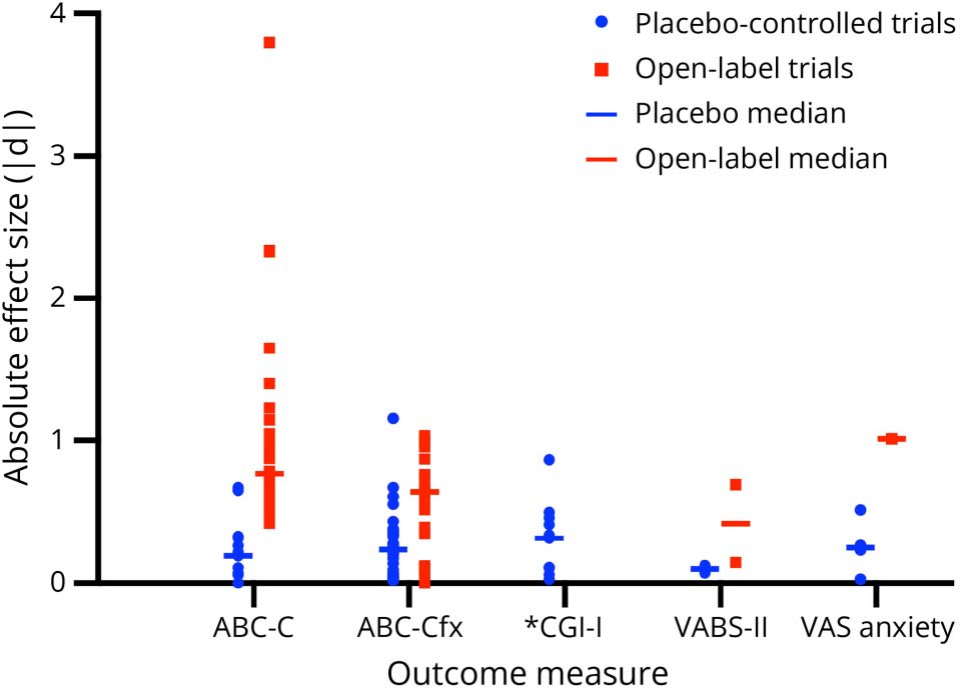
Distribution of Absolute Effect Sizes Across Outcome Measures in Placebo-Controlled and Open-Label Trials in Fragile X Syndrome Each point represents an individual absolute effect size (|Cohen's d|) extracted from placebo-controlled trials (blue) or open-label trials (red). Horizontal bars denote the median absolute effect size for each trial type within each outcome measure. Absolute effect sizes were used to allow comparison of the magnitude of treatment-related change regardless of direction. Medians were calculated separately for each outcome measure; for the ABC-C and ABC-Cfx instruments, all total and subscale scores were included when computing the median values to represent the overall responsiveness of each outcome measure. Larger median absolute effect sizes indicate greater observed magnitude of change. The figure was generated using GraphPad Prism version 10.6. *Effect size for CGI-I could not be computed for open-label trials because this measure lacks a baseline value. For each outcome measure, N indicates the number of studies contributing effect-size estimates for placebo-controlled vs open-label trials: ABC-C (N = 4 vs N = 5), ABC-Cfx (N = 8 vs N = 3), CGI-I (placebo only: N = 10), VABS-II (N = 1 vs N = 2), and VAS Anxiety (N = 4 vs N = 1). CGI-I = Clinical Global Impressions–Improvement; VABS-II = Vineland Adaptive Behavior Scales–Second Edition.

Overall, both open-label and placebo-controlled trials demonstrated variable treatment effects across outcome measures. Figure 5 displays the distribution of absolute effect sizes (|Cohen d|) extracted from each study and outcome measure, plotted separately for placebo-controlled and open-label trials. Absolute effect sizes were used to summarize the magnitude rather than the direction of change because the included trials evaluated heterogeneous pharmacologic agents with differing expected directions of effect, making signed estimates less interpretable. Effect sizes were consistently larger in open-label trials than in placebo-controlled trials across all measures. Across all studies and outcome measures, the overall median absolute effect size was 0.70 for open-label trials compared with 0.22 for placebo-controlled trials, indicating smaller treatment-related changes when placebo controls were implemented. These differences were most pronounced for the ABC-C and VAS Anxiety measures.

## Discussion

While the development of effective treatments for RDs such as FXS remains a priority for both researchers and individuals with lived experience, our study highlights several opportunities to improve future trial designs and reporting to improve detection of treatment efficacy. A central interpretive constraint of our synthesis is that pooled effect sizes predominantly index recurrent reported outcome measures that were typically secondary end points, while safety was most often the primary outcome measure. Consequently, studies may not have been powered sufficiently to show their full potential.

A recurring statistical limitation identified in the existing literature is the frequent omission of key statistical details necessary for the independent calculation of effect sizes. In particular, inconsistent reporting of key data such as within-group mean change scores, SDs of change, and preintervention and postintervention sample sizes limited the inclusion of studies. Of the 64 studies screened at the full-text stage, 25 were excluded due to insufficient statistical reporting. In cases where it may not be feasible to calculate the effect size directly, it would be beneficial for studies to provide alternative statistical information such as the pre-post correlation coefficients, *t* value or *p* value from an independent *t* test comparing 2 independent means (treatment and placebo), analysis of variance (ANOVA) F statistic, mean square error, odds ratio, covariate correlation, CIs for the mean difference between groups, etc., that would allow for indirect estimation of Cohen d.^39^

To facilitate future meta-analyses, we recommend that journals require authors to report either the effect size or sufficient statistical information to allow for its calculation.

In addition, our study highlights several key trial design issues in clinical trials for individuals with FXS that are of broader relevance to RDs.

First, although RCTs are widely regarded as the gold standard for evaluating medical interventions, open-label trials remain common in FXS research. This is largely due to barriers such as limited participant availability, absence of registries, limited funding and geographical spread of patients. Nonetheless, efforts toward conducting RCTs should be prioritized as we found that the median effect size was substantially smaller in placebo-controlled trials (median |d| = 0.22) compared with open-label studies (median |d| = 0.70). Factors that underpin the placebo effect such as expectancy, rater bias, or regression to the mean can individually or collectively inflate observed improvements in placebo arms, particularly in trials that rely on subjective outcome measures.^40-42^ Incorporating placebo controls in future FXS trials would help isolate treatment-related change and reduce the risk of overestimating efficacy due to placebo-associated inflation.

Second, an important difference between trials in FXS and those in broader neurodevelopmental populations such as Attention-Deficit/Hyperactivity Disorder (ADHD), ASD, or ID is the relatively small number of individuals included in FXS studies. The trials reviewed included a mixture of small and larger studies. On average, FXS studies in this review included 64 participants (range: 6–314) compared with 101 for ASD risperidone trials or 146 for ADHD methylphenidate trials.^43,44^ Smaller sample sizes result in larger standard errors and wider 95% CIs, limiting the precision of estimated treatment effects. Larger trials did not consistently demonstrate larger effect sizes, suggesting that limited efficacy of the investigational compounds, rather than sample size alone, may contribute to the modest treatment effects observed.

Third, variability in outcome measures, combined with small sample sizes, made it difficult to compare or group studies. Substantial heterogeneity was observed across trials in the behavioral scales and versions employed. For example, some studies used the original ABC-C, while others used the Fragile X–specific version (ABC-Cfx). Similarly, versions of the VABS ranged from Vineland-I to Vineland-II and III, with differences in scoring approaches (e.g., raw vs standard scores). This heterogeneity limited the number of studies eligible for comparative analysis and hindered the ability to synthesize findings across trials conducted over time. Adopting standardized, FXS-specific outcome measures (such as ABC-Cfx) would, therefore, be important to improve cross-study comparability, reduce methodological heterogeneity and enhance sensitivity to detect clinically meaningful treatment effects. In our full-text screening, only 23 of 64 studies used similar well-established outcome measures which made comparing treatments across trials difficult. Nonetheless, our analysis revealed emerging patterns in which outcome measures appeared most responsive across trials. Indeed, ABC-C and ABC-Cfx subscales showed most changes, followed by VAS Anxiety, CGI-I, and the VABS composite score. Of interest as mentioned above, those overall effect sizes were magnified in the absence of placebo as reflected by the total effect size.

It is important to note that most studies demonstrated substantial variability across subdomains within individual outcome scales, with no intervention showing uniformly robust effects across all ABC-Cfx subscales. Certain treatments appeared to yield more pronounced benefits in specific behavioral domains; for example, BPN14770 showed greater improvements in Stereotypy and Hyperactivity than in other subscales (eFigure 3). This domain-specific pattern was also evident in open-label trials of lovastatin. These findings highlight the importance of evaluating treatment effects at the subscale level, as clinically meaningful improvements in targeted behaviors may occur even when overall composite scores remain modest. However, these observations should be interpreted with caution. Many outcome measures used in FXS trials, such as the ABC-Cfx and VABS, include multiple subdomains that are interrelated within each instrument, and testing across numerous subscales inherently increases the likelihood of spurious significant effects.^7^ Apparent selectivity for a given subscale (e.g., irritability or speech) may therefore reflect shared variance among related behavioral dimensions, rather than true domain-specific drug activity. Moreover, when total scale scores remain unchanged (for instance, ABC-Cfx total in lovastatin), improvements in isolated subscales may need to be interpreted with caution. Future studies should incorporate appropriate statistical corrections for multiple comparisons and use composite or mechanistically aligned outcomes to better distinguish meaningful treatment effects from statistical noise.

Our review brought to light some important considerations for future studies.

In our review, we focused on identifying which outcome measures may be most “movable” by drugs and therefore grouped different compounds within the same graphs; however, we did not imply that these compounds share a common mechanism of action. Many trials relied on nonspecific behavioral scales, which may not directly reflect the pharmacodynamic effects of mechanism-specific compounds. It remains unclear how modulation of very different signaling pathways could lead to changes in the same phenotype. One challenge is that the phenotypes tested are usually broad (e.g., behavior, anxiety) and therefore could involve multiple pathways. The inclusion of molecular or physiologic (e.g., EEG) biomarkers may help stratify patients and improve understanding of drug response in future studies.

Clinical trials that rely heavily on caregiver-reported outcomes are particularly vulnerable to placebo effects, subjectivity, and rater bias.^13^ Incorporating objective measures such as EEG, wearable sensors, actigraphy, or eye-tracking could strengthen construct validity by capturing treatment effects closer to underlying mechanisms.^45^ These digital tools can detect subtle, continuous changes in activity, sleep, attention, or social engagement that may be missed in periodic assessments, particularly in minimally verbal populations. However, implementation remains challenging due to variability in tolerance and sensory sensitivities and the need to standardize hardware and software across platforms. Although identifying reliable, observable, and responsive biomarkers remains a challenge in FXS research, the incorporation of such objective measures could help mitigate placebo effects and improve the interpretability of caregiver-based outcome data.

Natural history studies play a critical role in validating emerging objective and digital end points, such as EEG-based biomarkers, wearable sensors, and actigraphy-derived measures. Longitudinal observational data allow researchers to establish baseline trajectories of behavioral and physiologic change in FXS, which are essential for differentiating true drug-related effects from normal developmental variability.^46^ In RDs, where sample sizes are small, such studies also help refine eligibility criteria, identify sensitive outcome windows, and support regulatory qualification of new end points. Greater alignment between interventional trials and natural history research will be essential to accelerate therapeutic development.

When designing RD trials, inclusion criteria, especially age range, require careful consideration. Considerable heterogeneity in participant age was observed across FXS studies, with some enrolling children, adolescents, or mixed-age cohorts. While often necessary given the rarity of FXS, this variability can affect treatment response. Trials involving pediatric participants generally reported larger effect sizes, whereas no adult-only studies yielded statistically significant results. Notably, all placebo-controlled studies that yielded statistically significant results included pediatric populations. For example, the minocycline and cannabidiol trials, all limited to children or adolescents, showed significant improvements.^23,30^ These findings underscore the potential importance of early intervention, and future studies should examine the long-term impact of early treatment on symptom progression in FXS. Distinct developmental stages, baseline behaviors, and adaptive functioning likely influence responsiveness to treatment. Clinical heterogeneity such as differences in FMR1 methylation, cognitive ability, and co-occurring autism or anxiety, may further modulate outcomes and contribute to inconsistent results. In addition, the psychometric sensitivity of outcome measures may vary by age, meaning a score change in a child may not equate to the same change in an adult. Adolescents and adults receiving placebo have also shown substantial improvements on caregiver-reported outcomes.^13^ To address these issues, future trials should consider stratified randomization, age-based subgroup analyses, or narrower inclusion criteria to enhance developmental homogeneity and improve the interpretability and precision of treatment effects.

### Limitations and Future Directions

A limitation of this review is the assumption of a correlation coefficient of 0.7 in cases where individual-level data, SDs of change scores, or author-supplied information were unavailable. This assumption introduces potential uncertainty and bias if the true pre-post correlation was substantially different than 0.7, which could potentially affect the effect sizes and CIs. To enable more precise calculations, future studies should report individual-level statistics such as the SDs of change scores and the correlation between baseline and follow-up measurements.

Another limitation is the exclusion of gray literature such as conference abstracts and trial registries due to limitations in quality appraisal and the frequent absence of detailed statistical information (SDs, change scores) required to compute standardized effect sizes. Although this may introduce publication bias, our primary aim was to perform effect size calculations, which could not be reliably derived from these sources.

Another limitation of this review is the heterogeneity among included studies. Substantial variation in methodology, study design, outcome measures, and treatment protocols likely contributed to the wide range of reported effect sizes. To reduce this, we grouped the studies into those with placebo groups and those without placebo groups (open-label studies).

Owing to the limited number of placebo-controlled studies, small sample sizes, and substantial heterogeneity, we were unable to draw definitive conclusions about the efficacy of pharmacologic treatments for FXS. Although several drugs showed promise in specific outcome domains, none of the treatments consistently demonstrated strong or robust effects across all measures.

Building on this scoping review, an ensuing systematic review could quantitatively synthesize effect sizes across pharmacologic classes, stratified by mechanism of action, participant age, and outcome type (reported vs objective). It should also incorporate gray literature, unpublished trials, and emerging digital or physiologic measures to reduce publication bias and capture early-phase innovation. Such an analysis would help clarify dose–response relationships, age-specific efficacy patterns, and the translational alignment between mechanistic targets and measurable outcomes. However, achieving consensus on which outcome measures best reflect meaningful clinical change remains a major challenge in FXS and other NDDs. Greater standardization across behavioral and functional end points will be critical to prevent the lack of overlap observed in previous trials and enable more reliable cross-study synthesis. Ultimately, this next step would bridge the descriptive mapping we provide here with a more definitive assessment of efficacy trends in FXS pharmacotherapy.

Nonetheless, these limitations highlight critical areas for improvement in future trial design. An emerging area of promise in FXS and other RDs lies in precision medicine and gene-editing technologies.^47^ Promising treatments undergoing research include SPG601, a novel synaptic therapy, and antisense oligonucleotide therapy.^48,49^ Given the need for larger, well-controlled clinical trials, it is essential that clinicians, researchers, and families collaborate to advance research in RDs and to develop effective, personalized treatment strategies.

## Glossary

ABC-C: aberrant behavior checklist-community
ASD: autism spectrum disorder
CGI-I: clinical global impressions–improvement
FMRP: fragile X mental retardation protein
FXS: fragile X syndrome
ID: intellectual disability
NDD: neurodevelopmental disability
RCT: randomized controlled trial
RD: rare disorder
VABS-II: vineland adaptive behavior scales–second edition
VAS: visual analog scale
